# Diagnostic value of blood culture growth patterns in distinguishing contaminants from pathogens

**DOI:** 10.1128/jcm.01210-25

**Published:** 2026-01-22

**Authors:** Eli Ben-Chetrit, Yigal Helviz, Phillip D. Levin

**Affiliations:** 1 Infectious Diseases Unit, Shaare Zedek Medical Center, The Eisenberg R&D Authority, Hebrew University School of Medicine 162914 , Jerusalem, Israel; 2 Department of Intensive Care Medicine, Shaare Zedek Medical Center, The Eisenberg R&D Authority, Hebrew University School of Medicine 162914 , Jerusalem, Israel; Cleveland Clinic, Cleveland, Ohio, USA

**Keywords:** discordant sets, concordant sets, contamination, true bacteremia

## Abstract

Blood culture contamination is common, causing diagnostic uncertainty and unnecessary antibiotic use. Analyzing growth patterns within culture sets might offer diagnostic value. We retrospectively analyzed peripheral blood culture sets from 2019 and 2024. Growth pattern (one bottle [discordant] vs both bottles [concordant]) was analyzed according to clinical significance (contaminant vs true pathogen). Overall, 38,216 blood culture sets were analyzed, including 1,491 (3.9%) discordant and 1,938 (5.1%) concordant sets (remaining 34,787 [91.0%] sets were sterile). Discordant sets grew 1,060/1,491 (71.1%) contaminants and 431/1,491 (28.9%) true pathogens. Concordant sets grew 222/1,938 (11.4%) contaminants and 1,716/1,938 (88.5%) true pathogens (*P* < 0.001). Examining coagulase-negative staphylococci (CoNS) only (2019 data set), 629/642 (98.0%) discordant sets grew contaminants, while 13/642 (2.0%) grew true pathogens. In contrast, *Staphylococcus aureus* grew in only 82/270 (30.4%) discordant sets. Among 858 first CoNS-positive cultures per patient, 624/636 (98.1%) discordant sets grew contaminants, and 12/636 (1.9%) grew CoNS defined as a true pathogen. The negative predictive value of a discordant first CoNS set to exclude true CoNS bacteremia was 98.1% (95% confidence interval 96.7%–98.9%). Examining aerobic vs anaerobic bottles in 356 discordant sets, contaminants were found more frequently in aerobic bottles (135/356, 37.9% vs 73/356, 20.5%, *P* = 0.04). The proportion of true pathogens was similar in both (79/356, 22.2% vs 69/356, 19.4%, *P =* 0.4). Discordant CoNS-positive cultures were strongly associated with contamination. This could assist in interpreting blood culture results and supporting antimicrobial stewardship. Discordance might result from a diversion effect, the aerobic bottle acting as a diversion device for the anaerobic bottle.

**IMPORTANCE:**

Rapidly distinguishing blood culture contaminants from true pathogens is essential for optimizing antimicrobial stewardship and avoiding unnecessary antibiotic therapy. In this large, two-period study, we demonstrate that discordant growth of coagulase-negative staphylococci in a two-bottle set has a negative predictive value of 98.1% for true bacteremia. This finding remained robust across both study years and when restricted to first positive cultures, highlighting its reliability. Incorporating simple growth pattern analysis into early blood culture interpretation can provide clinicians with reliable and timely information within 24 hours, supporting more targeted and judicious antibiotic use.

## INTRODUCTION

Blood culture contamination (BCC) by nonpathogenic bacteria, particularly coagulase-negative staphylococci (CoNS), can lead to diagnostic uncertainty, unjustified antibiotic administration, and increased healthcare costs (1–3). BCC is usually defined clinically based on the type of bacteria grown (specific commensal organisms) and by comparing results of multiple blood culture sets—growth in a single set among multiple sterile sets usually reflecting contamination. However, clinical context (such as the presence of foreign bodies, e.g., heart valves, pacemaker leads, or central lines) is also relevant.

Classification of a positive blood culture set as a contaminant often begins with the identification of gram-positive cocci on Gram stain. Rapid species identification methods, including rapid plasma coagulase testing (performed directly on samples from positive blood culture bottles), molecular assays, or direct matrix-assisted laser desorption/ionization time-of-flight mass spectrometry identification from the blood culture bottle, can be performed within a few hours of blood culture positivity potentially differentiating between *Staphylococcus aureus* and CoNS. Despite this, definitive identification and determination of susceptibility profiles typically require 48 to 72 hours.

If, alongside early Gram stain and rapid identification methods, the growth pattern within a given culture set (e.g., positivity in only one vs both bottles) could be demonstrated to accurately predict the presence of a contaminant, the clinician’s ability to distinguish true infection from contamination might be improved. This, in turn, could help reduce unnecessary antimicrobial use and associated healthcare costs.

We hypothesized that the growth of a potential contaminant organism (as defined by Gram stain and coagulase testing) in only one bottle of a blood culture set—a discordant set—could serve as a reliable predictor of BCC. This criterion could aid in reducing unnecessary antibiotic therapy by distinguishing true infections from contaminants more effectively.

## MATERIALS AND METHODS

**Fig 1:**
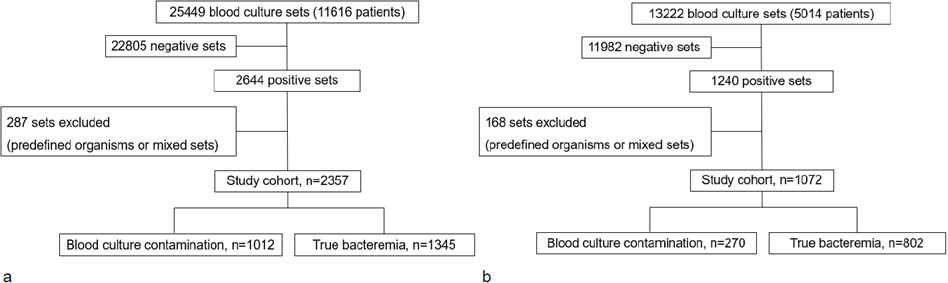
Derivation of study cohorts: 2019 (**a**) and 2024 (**b**).

Shaare Zedek Medical Center (SZMC) is a 1,000-bed academic referral hospital in Jerusalem, Israel. All blood cultures obtained by direct venipuncture (whether by needle, or at insertion of a peripheral intravenous (IV) catheter, but not from an existing line) from adult patients (aged 20 years or older) in SZMC during 2019 and 2024 were retrospectively included (Fig. 1).

At SZMC, blood culture sets are typically collected using Vacutainer equipment and consist of one aerobic and one anaerobic bottle. Until 2020, standard BD BACTEC Plus Aerobic/F and Anaerobic/F media (Becton Dickinson, USA) were used. Beginning in 2021, the anaerobic bottle was replaced with the BD BACTEC Lytic/10 Anaerobic/F bottle, while the Aerobic/F bottle was retained. In 2019, blood cultures were obtained without an initial specimen diversion device (diversion tube), whereas use of a diversion tube (as a means to reduce BCC) was recommended and widely adopted in 2024 (4). To minimize potential bias associated with the diversion collection technique, blood cultures obtained using diversion during 2024 (labeled as “diverted” in the electronic database) were excluded from the study data set.

Data for each bottle of each blood culture set (date, result, and aerobic/anaerobic for the 2024 data set) were extracted from the computerized microbiology database. Each isolate was classified as either a true pathogen or contaminant. Common skin commensals (e.g., CoNS, *Micrococcus* spp., *Corynebacterium* spp., *Bacillus* spp., and viridans group streptococci) were considered as contaminants unless one of the following criteria was met defining them as true pathogens: (i) repeated growth of the same organism in additional blood culture sets within a week *and* clinical evidence of infection on chart review (e.g., infective endocarditis); (ii) growth in both peripheral and central line cultures, suggesting central-line associated blood stream infection (CLABSI) (results of cultures taken from central lines were examined for this purpose only and not included in other analyses); and (iii) a single positive culture and administration of appropriate antibiotics for more than 72 hours in the relevant clinical context. Chart review was performed by E.B.-C.

Blood cultures obtained from sources other than peripheral venipuncture (e.g., subclavian, jugular, femoral, or long-term catheters such as PICC lines, Hickman, or Port-a-Cath) were excluded from the numerical analyses, due to the higher risk of colonization with CoNS or other skin commensals. However, their results (along with clinical context from chart review) were considered in defining whether a skin commensal was a true pathogen or not. In contrast, blood cultures drawn through newly inserted peripheral intravenous catheters were included, as colonization is not a concern in this setting. Although drawing cultures from peripheral IVs is generally discouraged and not recommended by our institutional protocol, it frequently occurs in “real-life” practice.

All bacteria not defined as contaminants as above were considered true pathogens with the following exceptions. (i) Obligate anaerobes, such as *Bacteroides* spp., *Clostridium* spp., *Prevotella* spp., *Veillonella* spp., *Fusobacterium* spp., *Campylobacter* spp., and *Cutibacterium* spp., were excluded as they would be expected to grow in only the anaerobic bottle. (ii) *Pseudomonas aeruginosa* and *Sphingomonas* spp. were excluded due to their preference for aerobic conditions. (iii) *Staphylococcus lugdunensis* was excluded due to its distinct pathogenic potential, which is more comparable to *S. aureus* than to other CoNS species. (iv) Sets with mixed growth (contaminants and true pathogens) were also excluded. Data were analyzed at the level of blood culture sets, each consisting of two bottles. Each blood culture set was defined as concordant if both bottles grew the same organism and discordant if only one bottle was positive. After the definition of each isolate as either a contaminant or true pathogen, the proportion of discordant versus concordant sets was compared between events classified as BCC and true bacteremia.

Three subgroup analyses were performed: (i) a comparison of concordant and discordant cultures limited to cultures growing only *S. aureus* (true pathogen) or CoNS (common contaminant) (these bacteria are expected to have similar growth characteristics in blood cultures—same genus organisms are unlikely to be affected by medium type); (ii) a patient-specific analysis including only the first positive blood culture obtained from each patient (whether contaminant or true pathogen) to reduce repetition bias from repeated positive blood cultures in the same patient; and (iii) an analysis of the results of the aerobic vs anaerobic bottle from discordant sets. This analysis was performed on the first positive culture in the 2024 data set.

The negative predictive value (NPV) of a first blood culture set growing CoNS isolates in only one bottle (a discordant set) was calculated. All comparisons of proportions were performed using the *χ* or Fisher’s exact test, as appropriate. Statistical analyses were performed using WINPEPI version 11.65, and a two-tailed *P*-value < 0.05 was considered statistically significant.

## RESULTS

**Fig 2:**
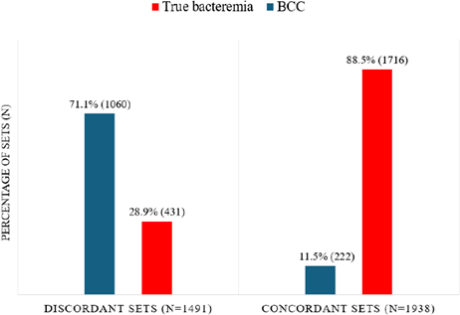
Discordant and concordant sets growing blood culture contaminants (BCC) and true pathogens (combined data sets 2019 and 2024). Discordant sets grew significantly more contaminants and significantly fewer true pathogens when compared to concordant sets (*P* < 0.001).

A total of 38,671 peripheral blood culture sets were evaluated, including 25,449 sets obtained during 2019 and 13,222 sets during 2024. The number of sets in 2024 was lower due to exclusion of sets taken following use of a diversion device. An additional 287 sets were excluded from the 2019 dataset and 168 from the 2024 data set leaving 38,216 sets for analysis (Fig. 1). Of these, 34,787 (91.0%) were sterile, 1,491 (3.9%) discordant, and 1,938 (5.1%) concordant. Discordant sets grew 1,060/1,491 (71.1%) contaminants and 431/1,491 (28.9%) true pathogens, while concordant sets grew 222/1,938 (11.4%) contaminants and 1,716/1,938 (88.5%) true pathogens (Fig. 2). Discordant sets grew significantly more contaminants and significantly fewer true pathogens when compared to concordant sets (*P* < 0.001). These differences remained significant when considering only 2019 or 2024 data sets (Tables S1 and S2). The overall contamination rate decreased significantly from 2019 to 2024 (1,012/25,162 [4.0%] vs 270/13,054 [2.1%, *P* < 0.001], while the proportion of sets growing a true pathogen increased (1,345/25,162 [5.3%] vs 802/13,054 [6.1%], *P* < 0.001).

A subgroup analysis was performed comparing only sets growing CoNS and *S. aureus*—bacteria of the same genus that were assumed to have identical growth patterns in culture media. This analysis was limited to cultures obtained in 2019. CoNS were identified in 852 culture sets, of which 642/852 (75.3%) sets were discordant and 210/852 (24.7%) concordant. CoNS was defined as a contaminant in 795/852 (93.3%) sets and as a true pathogen in 57/852 (6.7%) sets. Among discordant sets, 629/642 (98.0%) grew contaminants, and 13/642 (2.0%) sets grew true pathogens. Considering all 57 sets growing CoNS defined as a true pathogen, 13/57 (22.8%) were discordant.

**Fig 3:**
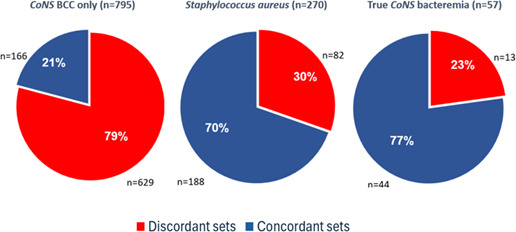
Discordance patterns across CoNS BCC, *S. aureus* bacteremia, and true CoNS bacteremia (2019 data set). Among discordant sets growing CoNS, 629/642 (98.0%) grew contaminants and 13/642 (2.0%) sets grew true pathogens (*P* < 0.001). The proportion of discordant sets among *S. aureus* isolates was similar to that observed in true CoNS bacteremia (82/270 [30%] vs 13/57 [23%], *P* = 0.34).

All 270 cultures growing *S. aureus* were considered as true pathogens and included 82/270 (30.4%) discordant sets and 188/270 (69.6%) concordant sets. The proportion of discordant sets growing CoNS defined as a true pathogen (13/642, 2.0%) was significantly lower than the proportion of discordant sets growing *S. aureus* (82/270, 30.0%, *P* < 0.001). In contrast, the proportion of the 57 sets growing CoNS defined as a true pathogen that were discordant (13/57, 23.0%) was similar to the proportion of sets growing *S. aureus* that were discordant (82/270, 30.0%, *P* = 0.34) (Fig. 3).

**Fig 4:**
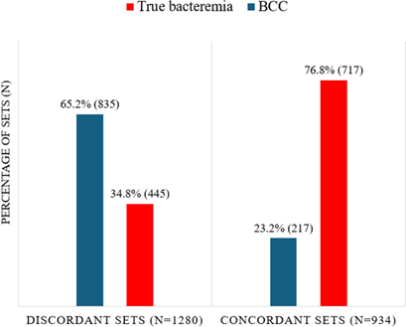
Discordant and concordant sets growing first positive blood culture contaminants (BCC) vs true pathogens. Discordant sets grew significantly more contaminants and significantly fewer true pathogens when compared to concordant sets (*P* < 0.001).

A second subgroup analysis was performed including only the first positive culture per patient (contaminant or true positive) to control for bias related to multiple positive cultures in the event of true bacteremia. This analysis included 2,214 culture sets (from both 2019 and 2024), of which 1,280/2,214 (57.8%) were discordant and 934/2,214 (42.2%) concordant. Overall, 1,052/2,214 sets (48.0%) grew a contaminant, and 1,162/2,214 (52.0%) sets grew a true pathogen. Discordant sets grew a contaminant on 835/1,280 (65.2%) occasions vs a true pathogen on 445/1,280 (34.8%) occasions (*P* < 0.001) (Fig. 4; Tables S3 and S4).

Considering only the 858 first positive sets per patient growing CoNS isolates, these were discordant on 636/858 (74.1%) and concordant on 222/858 (24.9%) occasions. Contaminants grew in 819/858 (95.0%) sets and true pathogens in the remaining 39/858 (5.0%) sets. Among discordant sets, 624/636 (98.1%) grew contaminants, and 12/636 (1.9%) grew CoNS defined as a true pathogen (Table 1). Notably, 9/12 (75.0%) patients with a discordant culture and true CoNS bacteremia had either device-related infections or endocarditis. The NPV of a discordant first CoNS set to exclude true CoNS bacteremia was 98.1% (95% confidence interval 96.7%–98.9%).

**TABLE 1:** Discordant and concordant sets among first positive culture sets with coagulase-negative staphylococci

First-CoNS isolates	Discordant sets	Concordant sets	Total
True CoNS bacteremia (*n*, row %)	12 (30.8%)^*a*^	27 (69.2%)	39
Column %	1.9%	12.2%	
BCC (*n*, row %)	624 (76.2%)	195 (23.81%)	819
Column %	98.1%	87.8%	
Total	636	222	858

^*a*^ Across the 2019 and 2024 datasets, 12 patients had true-positive first-CoNS discordant sets. Seven had catheter-related bloodstream infection, one had prosthetic valve endocarditis, one had endocarditis related to intravenous drug use, one had a deep soft tissue infection, one had post-instrumentation urosepsis, and the source remained unidentified in one case.

**Fig 5:**
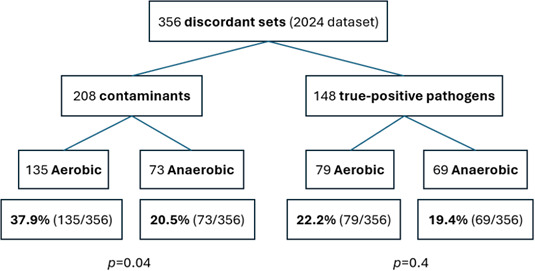
Bottle type analysis (aerobic vs anaerobic) and culture outcome in 356 discordant sets (2024 data set).

The final subgroup analysis examined the correlation between bottle type (aerobic vs anaerobic) and culture outcome in discordant sets. This analysis was limited to the first positive set from the 2024 data set. Among 356 discordant culture sets that grew a bacterium, in cases where only the aerobic bottle was positive, there were 79/356 (22.2%) true pathogens and 135/356 (37.9%) contaminants. In contrast, if only the anaerobic bottle was positive, there were 69/356 (19.4%) true pathogens and 73/356 (20.5%) contaminants. The proportion of true pathogens in the aerobic vs anaerobic bottle was similar (*P* = 0.4), while the proportion of contaminants was significantly lower in the anaerobic bottle (*P* = 0.04) (Fig. 5). Examining the six first positive CoNS cultures taken in 2024 that grew in discordant sets and were associated with *true* infection, five grew in the aerobic bottle only and one in the anaerobic bottle only.

## DISCUSSION

In this large study, we have demonstrated a strong association between discordant growth of organisms usually defined as contaminants in a blood culture set (growth in a single bottle only) and the absence of defined infection. In other words, upon receipt of an initial blood culture report indicating growth of a coagulase-negative staphylococcus in a single bottle of a blood culture set, there is a 98.0% chance that this reflects BCC and requires no further action. Needless to say, the results of additional blood cultures and the clinical constellation must be considered, particularly in patients at high risk for CoNS bacteremia such as those with prosthetic hardware.

The finding that discordant sets are associated with contamination is robust and supported by three subgroup analyses. The first subgroup analysis included a comparison of only CoNS to *S. aureus*. These bacteria are from the same genus and expected to have similar growth characteristics in culture media. The incidence of discordant sets was high for CoNS and low for *S. aureus* (always considered to be a true pathogen). This implies that it was not differential growth in aerobic vs anaerobic media that caused contaminants to grow in only one bottle while true pathogens grew in both (Fig. 3) but rather contamination vs infection.

In the second subgroup analysis, we controlled for a repeated culture bias by including only the first positive set per patient regardless of definition. True bacteremia is often found in multiple sets taken around a suspected septic episode. As true bacteremia was expected to be associated with a lower incidence of discordant culture sets, this could bias the comparison with contaminated blood cultures (which are often isolated sets)—potentially increasing the proportion of sets with concordant growth (true pathogens) relative to discordant growth (contaminants). As anticipated, this analysis revealed a greater difference in the proportion of discordant sets associated with contaminants vs true pathogens, further supporting the study hypothesis (Fig. 4) and the negative predictive potential (for infection) of a discordant set growing a potential contaminant (Table 1).

The third subgroup analysis examined the results of aerobic vs anaerobic culture bottles in discordant sets. Contaminants are usually found in the first aliquots of blood taken from the vein. Growing evidence suggests that diversion of the initial blood specimen can significantly reduce BCC by diverting skin bacteria that survive skin surface disinfection (4–7). Both initial specimen diversion devices and simpler lithium-heparin diversion tubes have demonstrated efficacy in lowering contamination rates (7, 8). The hospital protocol states that the aerobic bottle should be taken first, and thus, it was expected that most contaminants would be found in the aerobic bottle and not the anaerobic bottle. Among discordant contaminant sets, anaerobic bottles were positive only half as often as aerobic bottles (20.5% versus 37.9%). Thus, our results appear consistent with the diversion concept: the first bottle in a set may function as an unintended diversion device for the second, thereby reducing contamination within the pair. It must be noted that while hospital protocol mandates filling the aerobic bottle first, there was no measure of compliance in this study.

Importantly, this study focused on potential contaminants and not bacteria defined as true pathogens, such as gram-negative bacteria. While there were discordant sets growing such bacteria, they are identified early by Gram stain and do not present a therapeutic dilemma. Growth of a gram-negative bacteria in even one bottle provides sufficient indication to begin antibiotic therapy.

Our findings align with earlier reports. In a prospective study of a single-sampling strategy (SSS), an association between the number of positive bottles within a six-bottle set and the clinical significance of the organism has been demonstrated. Most often, contaminants were isolated from the first (>90%) or second bottles. The CoNS bloodstream infection rate positively correlated with the number of positive bottles (9). Similarly, Osaki et al. (10) analyzed four-bottle (two-set) collections and found that a single positive bottle—or two positives confined to one set—had a 100% NPV for true CoNS bacteremia. Both studies, however, differ from standard current practice: Leyssene’s SSS approach with six bottles is rare today, and Osaki’s work focused on multipleset patterns. In addition, the sample size in the Osaki study was small (*n* = 206), and the rate of true CoNS bacteremia was surprisingly high (30%–40%), perhaps limiting the generalizability of the findings. Our analysis adds to this literature by focusing on the diagnostic performance of the first positive culture—a clinically relevant scenario where therapeutic decisions are often made before subsequent blood culture results become available.

While the aforementioned studies primarily addressed laboratory parameters, Beekmann et al. (11) provided an important complementary clinical perspective, emphasizing host and systemic factors in predicting true CoNS bacteremia. Their algorithm identified true infection when at least two cultures were positive for CoNS within five days, or when a single positive culture was accompanied by clinical signs of infection. Consistent with our results, 95% of CoNS isolates classified as contaminants in their study were positive in only one culture. In contrast, our analysis centered on the diagnostic value of the first CoNS-positive set and specifically the NPV of discordant results. This approach provides a practical tool for real-time decision-making, integrating laboratory data with bedside context rather than relying solely on cumulative culture results. The proportion of true CoNS bacteremia in our cohort (4.5% [39/858] [Table 1]) was lower than in Beekmann’s multicenter study (22.0%), likely reflecting differences in patient populations and the exclusion of central line-drawn cultures in our analysis. Nonetheless, the presence of central lines among some peripheral CoNS bacteremia cases in our cohort highlights the importance of clinical context in decision-making.

Our study has several strengths, including a large data set of blood culture sets, with high statistical power and the reproducibility of our findings in two distinct time periods. Critically, our methodology addresses potential culture media bias by excluding organisms with strict aerobic or anaerobic requirements, by comparing within the same genus (CoNS versus *S. aureus*), by avoiding repeated positive bias for true pathogens and by examining the distribution of positive sets among aerobic and anaerobic bottles within the study groups, thereby minimizing the influence of bottle type on the results.

A significant limitation of the study is the absence of data on the exact blood volume inoculated into each bottle (blood volume can strongly influence culture yield). Prior studies have shown that the second bottle in a set is typically inoculated with a slightly lower volume - approximately 1 mL on average (12, 13). This might increase the proportion of discordant sets. However, this factor is unlikely to explain our findings. The similar proportions of discordant sets among *S. aureus* and true CoNS bacteremia, as well as the comparable recovery of true pathogens in aerobic (79/148) and anaerobic (69/148) bottles from discordant sets (Fig. 5), suggest that our observations reflect biological and clinical factors rather than technical variability related to blood volume.

Additionally, a subset of cultures was drawn via peripheral intravenous catheters at the time of their insertion, possibly elevating contamination risk, though this was not documented. While we rigorously reviewed cases suspected of true CoNS bacteremia, it remains possible that subclinical true infections were inadvertently classified as contaminants. Importantly, the use of NPV, unlike sensitivity, depends on prevalence and pretest probability; hence, it may be irrelevant among patients with foreign bodies (central venous catheters) or in case of suspected endovascular infection (i.e., presence of a prosthetic valve or a pacemaker). Finally, we did not analyze time-to-positivity data - a metric that has been shown to help differentiate contamination from true bacteremia (14–16).

To summarize, our findings suggest that receipt of a discordant CoNS-positive blood culture has a high NPV, indicating a very low risk of true CoNS bacteremia in many patients. This approach should complement, not replace, clinical judgment particularly in patients with indwelling devices such as prosthetic valves, pacemakers, or central venous catheters, or in those with postoperative or deep-seated infections. In these situations, CoNS isolation should not be dismissed as contamination. Conversely, when discordant CoNS growth is identified in patients with a low likelihood of true infection, antibiotic therapy may be withheld, as these findings most often reflect contamination. Interpretation should remain individualized, balancing diagnostic stewardship with the risk of undertreatment.

## AFTER EPUB

*[This article was published on 22 January 2026 with spelling errors in author names. The errors were corrected in the current version, posted on 23 January 2026.]*

## SUPPLEMENTAL MATERIAL

Tables S1 to S4 jcm.01210-25-s0001.docx Concordant vs discordant blood culture sets for 2019 and 2024 data sets.

ASM does not own the copyrights to Supplemental Material that may be linked to, or accessed through, an article. The authors have granted ASM a non-exclusive, world-wide license to publish the Supplemental Material files. Please contact the corresponding author directly for reuse.
