# Supplementary material for: Diagnostic value of blood culture growth patterns in distinguishing contaminants from pathogens
Source: J Clin Microbiol. 2026 Jan 22;64(2):e01210-25. doi: 10.1128/jcm.01210-25 (PMC12892935; doi:10.1128/jcm.01210-25)
Supplement: Tables S1 to S4 — Concordant vs discordant blood culture sets for 2019 and 2024 data sets. [file jcm.01210-25-s0001.docx]

## Supplemental tables

| 2019 | **Concordant** | **Discordant** | Total |
| --- | --- | --- | --- |
| **Contaminants (%)** | 184 (18.2%) | 828 (81.8%) | 1012 |
| Column % | 17.9% | 62.3% |  |
| **True pathogen (%)** | 843 (62.68%) | 502 (37.3%) | 1345 |
| Column % | 82.1% | 37.7% |  |
| Total | 1027 | 1330 | 2357 |
| Sterile cultures 22805 of 25162 (90.6%) | | | |

Supplemental Table 1. Concordant vs discordant blood culture sets; 2019 dataset

| 2024 | **Concordant** | **Discordant** | Total |
| --- | --- | --- | --- |
| **Contaminants (%)** | 38 (14.1%) | 232 (85.9%) | 270 |
| Column % | 8.3% | 37.8% |  |
| **True pathogens (%)** | 421 (52.49%) | 381 (47.5%) | 802 |
| Column % | 91.7% | 62.2% |  |
| Total | 459 | 613 | 1072 |
| Sterile cultures 11982 of 13054 (91.8%) | | | |

Supplemental Table 2. Concordant vs discordant blood culture sets; 2024 dataset

| 2019 | Concordant | Discordant | Total |
| --- | --- | --- | --- |
| **Contaminants (%)** | 191 (23.35%) | 627 (76.7%) | 818 |
| Column % | 28.1% | 67.9% |  |
| **True pathogens (%)** | 488 (62.2%) | 297 (37.8%) | 785 |
| Column % | 71.9% | 32.1% |  |
| Total | 679 | 924 | 1603 |

## Supplemental table 3. First-positive concordant vs discordant blood culture sets; 2019 dataset

| 2024 | **Concordant** | **Discordant** | Total |
| --- | --- | --- | --- |
| **Contaminants (%)** | 26 (11.11%) | 208 (88.9%) | 234 |
| Column % | 10.2% | 58.4% |  |
| **True pathogens (%)** | 229 (60.7%) | 148 (39.3%) | 377 |
| Column % | 89.8% | 41.6% |  |
| Total | 255 | 356 | 611 |

Supplemental table 4. First-positive concordant vs discordant blood culture sets; 2024 dataset
